# Associations between antimicrobial susceptibility/resistance of *Neisseria gonorrhoeae* isolates in European Union/European Economic Area and patients’ gender, sexual orientation and anatomical site of infection, 2009–2016

**DOI:** 10.1186/s12879-021-05931-0

**Published:** 2021-03-18

**Authors:** Susanne Jacobsson, Michelle J. Cole, Gianfranco Spiteri, Michaela Day, Magnus Unemo, on behalf of The Euro-GASP Network, Claudia Eder, Sonja Pleininger, Alexander Indra, Steliana Huhlescu, Irith De Baetselier, Wim Vanden Berghe, Blaženka Hunjak, Tatjana Nemeth Blažić, Panayiota Maikanti-Charalambous, Despo Pieridou, Hana Zákoucká, Helena Žemličková, Steen Hoffmann, Lasse Jessen Schwartz, Rita Peetso, Jevgenia Epstein, Jelena Viktorova, Ndeindo Ndeikoundam, Beatrice Bercot, Cécile Bébéar, Florence Lot, Susanne Buder, Klaus Jansen, Vivi Miriagou, Georgios Rigakos, Vasilios Raftopoulos, Eszter Balla, Mária Dudás, Lena Rós Ásmundsdóttir, Guðrún Sigmundsdóttir, Guðrún Svanborg Hauksdóttir, Thorolfur Gudnason, Aoife Colgan, Brendan Crowley, Sinéad Saab, Paola Stefanelli, Anna Carannante, Patrizia Parodi, Gatis Pakarna, Raina Nikiforova, Antra Bormane, Elina Dimina, Monique Perrin, Tamir Abdelrahman, Joël Mossong, Jean-Claude Schmit, Friedrich Mühlschlegel, Christopher Barbara, Francesca Mifsud, Alje Van Dam, Birgit Van Benthem, Maartje Visser, Ineke Linde, Hilde Kløvstad, Dominique Caugant, Beata Młynarczyk-Bonikowska, Jacinta Azevedo, Maria-José Borrego, Marina Lurdes Ramos Nascimento, Peter Pavlik, Irena Klavs, Andreja Murnik, Samo Jeverica, Sandra Kosmac, Tanja Kustec, Julio Vázquez Moreno, Asuncion Diaz, Raquel Abad, Inga Velicko, Magnus Unemo, Gwenda Hughes, Jill Shepherd, Lynsey Patterson

**Affiliations:** 1 WHO Collaborating Centre for Gonorrhoea and other STIs, National Reference Laboratory for Sexually Transmitted Infections, Department of Laboratory Medicine, Faculty of Medicine and Health, Örebro University, Örebro, Sweden; 2 National Infection Service, Public Health England, Colindale, UK; 3 European Centre for Disease Prevention and Control, Stockholm, Sweden

**Keywords:** Gonorrhoea, Ceftriaxone, Azithromycin, Antimicrobial resistance, Surveillance, European Gonococcal Antimicrobial Surveillance Programme (Euro-GASP), Europe

## Abstract

**Background:**

The emergence and spread of antimicrobial resistance (AMR) in *Neisseria gonorrhoeae*, nationally and internationally, is a serious threat to the management and control of gonorrhoea. Limited and conflicting data regarding the epidemiological drivers of gonococcal AMR internationally have been published. We examined the antimicrobial susceptibility/resistance of gonococcal isolates (*n* = 15,803) collected across 27 European Union/European Economic Area (EU/EEA) countries in 2009–2016, in conjunction to epidemiological and clinical data of the corresponding patients, to elucidate associations between antimicrobial susceptibility/resistance and patients’ gender, sexual orientation and anatomical site of infection.

**Methods:**

In total, 15,803 *N. gonorrhoeae* isolates from the European Gonococcal Antimicrobial Surveillance Programme (Euro-GASP), 2009–2016, were examined. Associations between gonococcal susceptibility/resistance and patients’ gender, sexual orientation and anatomical site of infection were investigated using univariate and multivariate logistic regression analysis. Statistical significance was determined by Pearson χ^2^-test or Fisher’s exact test with two-tailed *p*-values of < 0.05 indicating significance.

**Results:**

The overall gonococcal resistance from 2009 to 2016 was 51.7% (range during the years: 46.5–63.5%), 7.1% (4.5–13.2%), 4.3% (1.8–8.7%), and 0.2% (0.0–0.5%) to ciprofloxacin, azithromycin, cefixime, and ceftriaxone, respectively. The level of resistance combined with decreased susceptibility to ceftriaxone was 10.2% (5.7–15.5%). Resistance to cefixime and ciprofloxacin, and resistance combined with decreased susceptibility to ceftriaxone were positively associated with urogenital infections and heterosexual males, males with sexual orientation not reported and females (except for ciprofloxacin), i.e. when compared to men-who-have-sex-with-men (MSM). Azithromycin resistance was positively associated with heterosexual males, but no association was significant regarding anatomical site of infection.

**Conclusions:**

Overall, sexual orientation was the main variable associated with gonococcal AMR. Strongest positive associations were identified with heterosexual patients, particularly males, and not MSM. To provide evidence-based understanding and mitigate gonococcal AMR emergence and spread, associations between antimicrobial susceptibility/resistance and patients’ gender, sexual orientation and anatomical site of infection need to be further investigated in different geographic settings. In general, these insights will support identification of groups at increased risk and targeted public health actions such as intensified screening, 3-site testing using molecular diagnostics, sexual contact tracing, and surveillance of treatment failures.

**Supplementary Information:**

The online version contains supplementary material available at 10.1186/s12879-021-05931-0.

## Background

Globally, 87 million new gonorrhoea cases among adults each year were estimated in 2016 [1], which represent an increase with 12% compared to the 78 million estimated cases in 2012 [2]. In the European Union/European Economic Area (EU/EEA), the incidence of reported gonorrhoea cases increased from 7.8 (cases per 100,000 population) in 2008 to 26.4 in 2018 [3]. Effective antimicrobial treatment together with appropriate prevention, diagnostics, notification and treatment of sexual contacts, including test of cure (TOC), and a detailed understanding of the epidemiology, are the mainstays in controlling gonorrhoea. However, high levels of antimicrobial resistance (AMR) in *Neisseria gonorrhoeae* seriously threaten the management and control of the infection [3–7]. The susceptibility to the last remaining effective options for empiric first-line monotherapy, i.e. the extended-spectrum cephalosporin (ESC) ceftriaxone given in combination therapy together with azithromycin or as monotherapy [8–16], is also decreasing and since 2015 an international spread of at least one ceftriaxone-resistant gonococcal strain has been documented [4–8, 17–26]. Regular and quality-assured antimicrobial susceptibility surveillance for *N. gonorrhoeae* is imperative, to monitor current and emerging trends in AMR and to ensure effective patient management on an individual level as well as by timely revisions of treatment guidelines. In the EU/EEA, the antimicrobial susceptibility surveillance is conducted through the European Gonococcal Antimicrobial Surveillance Programme (Euro-GASP), which is a sentinel surveillance programme co-ordinated by the European Centre for Disease Prevention and Control (ECDC) since 2009 [6, 7, 27–29]. From 2009 to 2012, the 2009 European gonorrhoea management guideline was for urethral, cervical and rectal gonorrhoea recommending treatment as follows: ceftriaxone 250 mg intramuscular (IM) as a single dose or cefixime 400 mg oral as a single dose or spectinomycin 2 g IM as a single dose. For pharyngeal infection, ceftriaxone 250 mg IM as a single dose was recommended [30]. From 2012, the 2012 European gonorrhoea management guideline recommended dual therapy with ceftriaxone 500 mg plus azithromycin 2 g for both urogenital and extragenital gonorrhoea [31].

In several countries, emergence and spread of AMR *N. gonorrhoeae* strains have been associated with the gender and in particular sexual orientation of the gonorrhoea patients. For example, the spread of gonococcal AMR has been documented in high-frequency transmitting groups at increased risks such as men who have sex with men (MSM), after which the AMR has bridged over to the heterosexual population resulting in large and rapid spread across the country [32–35]. In Australia, significant differences in levels of resistance and decreased susceptibility were identified from gonococcal isolates derived from males versus females for all antimicrobials assessed [36]. Furthermore, it was recently suggested that genomic adaptation of gonococcal strains to the cervical environment is associated with increased antimicrobial susceptibility [37]. Regarding anatomical site of infection, pharyngeal gonorrhoea has been more common among MSM and gonococcal resistance to β-lactam antimicrobials, including the ESCs, may emerge in the pharynx through the acquisition of genetic AMR determinants from non-gonococcal commensal Neisseria species. For instance, *N. cinerea* and *N. flavescens* have been shown to harbour resistance-mediating *penA* sequences that can be transferred to gonococcal strains and cause resistance to penicillin, ceftriaxone and cefixime [33, 38–40]. Pharyngeal gonorrhoea can also support the spread of gonorrhoea and gonococcal AMR since these infections are mostly asymptomatic (> 90%), less frequently detected, and substantially more difficult to eradicate than infections at urogenital or anorectal sites. Accordingly, pharyngeal gonorrhoea can act as a reservoir for both infection and emergence and spread of AMR [33, 41]. Nevertheless, limited and also conflicting data regarding the level of gonococcal antimicrobial susceptibility/resistance in different anatomical sites of infection, e.g. pharyngeal and rectal sites compared to urogenital sites, have been published [41–46].

The aim of the present study was to investigate associations between the antimicrobial susceptibility/resistance of gonococcal isolates collected in 27 countries across EU/EEA from 2009 to 2016 and patients’ gender, sexual orientation and anatomical site of infection.

## Methods

### European Gonococcal Antimicrobial Surveillance Programme (Euro-GASP)

Euro-GASP has been previously described in detail [6, 7, 27–29, 47–51]. Briefly, participating EU/EEA countries submit a sample of consecutive *N. gonorrhoeae* isolates (ideally 100–200 isolates dependent on the number of gonorrhoea cases reported annually in the country) from their routine diagnostics for centralised antimicrobial susceptibility testing (AST) or, if countries have fulfilled set quality assurance criteria, they perform decentralised AST in their own countries. Countries include only one isolate per patient from those who were infected multiple times within a 4-week period or at several anatomical sites, to represent different gonorrhoea episodes. When multiple isolates per patient and gonorrhoea episode, priority is given to test extragenital isolates, particularly pharyngeal ones [48]. For decentralised AST, countries should have performed consistently well in the annual mandatory Euro-GASP external quality assessment (EQA) [50] and have had a good comparability between the laboratories own national or regional AST data and AST data generated by centralised testing [48]. During 2009–2016, the number of Euro-GASP countries increased from 17 to 23 and the number of decentralised Euro-GASP countries increased from 12 to 17. Centralised AST is currently conducted using minimum inhibitory concentration (MIC; mg/L) gradient strip tests for ceftriaxone, cefixime, and azithromycin, and MIC gradient strip test or agar dilution breakpoint method, which does not provide MIC and only susceptibility category (using agar plates with concentrations of 0.03 mg/L and 0.06 mg/L), for ciprofloxacin. For quality control of the AST in Euro-GASP [48], the 2016 WHO *N. gonorrhoeae* reference strains G, K, M, P and O (only when spectinomycin is tested) [51] are used. Furthermore, all centralised and decentralised Euro-GASP countries participate in the mandatory annual Euro-GASP EQA [50]. The MICs of each antimicrobial are interpreted into resistant (R); susceptible, increased exposure (I; formerly intermediate susceptibility); or susceptible (S) using current clinical breakpoints recommended by the European Committee on Antimicrobial Susceptibility Testing (EUCAST; www.eucast.org/clinical_breakpoints/), i.e. ceftriaxone and cefixime (S ≤ 0.125 mg/L, R > 0.125 mg/L), and ciprofloxacin (S ≤ 0.032 mg/L, R > 0.064 mg/L). In 2019, EUCAST excluded their clinical breakpoints for azithromycin. However, because agar dilution breakpoint method was previously used by many Euro-GASP countries also for azithromycin and accordingly no MICs of azithromycin are available, in the present study, we had to use the previous clinical azithromycin breakpoints (S ≤ 0.25 mg/L; R > 0.5 mg/L). Due to the low number of isolates with ceftriaxone resistance, analysis was performed using isolates with resistance (MIC> 0.125 mg/L) combined with decreased susceptibility (DS; MIC> 0.032–0.125 mg/L) to ceftriaxone. In Euro-GASP, the AST and *N. gonorrhoeae* species identification should be repeated for all isolates that are resistant to ceftriaxone, have elevated resistance to cefixime (MIC> 0.25 mg/L), and all isolates showing high-level resistance to azithromycin (MIC≥256 mg/L). Those isolates are also recommended to be sent to the Euro-GASP Reference Laboratory Hub (Public Health England/Örebro University Hospital) for further verification and whole genome sequencing [48].

All countries participating in Euro-GASP report all results through The European Surveillance System (TESSy), a web-based reporting system managed by the ECDC, where in addition to antimicrobial susceptibility/resistance data also epidemiological and clinical data of the corresponding gonorrhoea patients are included. The collected epidemiological and clinical data include gender, age, country of origin, mode of transmission (referred to as sexual orientation below: females, heterosexual males and MSM), anatomical site of infection (urogenital, pharyngeal and anorectal), HIV status, previous gonorrhoea diagnosis, and probable country of infection [6, 7, 27–29, 47–50].

### Statistical analysis

Associations between resistance/decreased susceptibility and patients’ gender, sexual orientation and anatomical site of infection were investigated using univariate and multivariate logistic regression analysis with MSM as the reference for sexual orientation and urogenital for site of infection. To control for the different years, year of isolate collection, with 2009 as the base, was included in the multivariate analysis. The odds ratios (OR) and 95% confidence intervals (CI) were calculated and Pearson’s χ2 test was used to measure if these odds ratios differed significantly from 1. Using a forward step-wise approach, the most significant and strongest associations from the univariate analysis were added to a multivariable logistic regression model sequentially. The statistical significance was determined by Pearson χ^2^-test or by Fisher’s exact test if cell numbers were less than 5, considering two-sided *p*-values of < 0.05 indicating significance. Statistical analysis was performed in Stata 15.1 (StataCorp, Texas, USA).

## Results

### Euro-GASP data from 2009 to 2016

From 2009 to 2016, 15,803 *N. gonorrhoeae* isolates from 27 countries were examined in Euro-GASP. Austria, Belgium, Denmark, France, Germany, Greece, Italy, Latvia, Malta, the Netherlands, Norway, Portugal, Slovakia, Slovenia, Spain, Sweden, and the United Kingdom (17 countries) have participated in Euro-GASP since 2009, in 2010 four additional countries joined (Cyprus, Hungary, Ireland, and Romania), in 2013 Iceland, in 2014 Estonia and Poland, in 2015 Croatia, and finally in 2016 Czech Republic and Luxembourg.

The number of Euro-GASP isolates reported in 2009–2016, which significantly increased from 1366 isolates in 2009 to 2663 isolates in 2016, gender and sexual orientation of the corresponding patients, and anatomical site of infection have been summarised in Table 1.

**Table 1:** Total number and proportion (%) of *Neisseria gonorrhoeae* isolates collected in Euro-GASP from 2009 to 2016 divided into gender, sexual orientation and anatomical site of infection

	2009 No (%)	2010 No (%)	2011 No (%)	2012 No (%)	2013 No (%)	2014 No (%)	2015 No (%)	2016 No (%)	Total No (%)
**Total no of isolates**	1366 (8.6)	1766 (11.2)	1902 (12.0)	1926 (12.2)	1994 (12.6)	2151 (13.6)	2035 (12.9)	2663 (16.9)	15,803 (100)
**Gender and sexual orientation**									
Females	219 (16.3)	308 (17.6)	321 (17.6)	310 (16.3)	302 (15.3)	318 (14.9)	363 (18.0)	395 (14.9)	2536 (16.2)
Males_Hetero	314 (23.4)	426 (24.4)	423 (23.1)	389 (20.4)	376 (19.0)	485 (22.7)	419 (20.7)	632 (23.8)	3464 (22.2)
Males_MSM	251 (18.7)	395 (22.6)	442 (24.2)	408 (21.4)	496 (25.1)	594 (27.8)	657 (32.5)	696 (26.2)	3939 (25.2)
Males_Other/UNK	559 (41.6)	620 (35.4)	642 (35.1)	798 (41.9)	804 (40.6)	743 (34.7)	583 (28.8)	931 (35.1)	5680 (36.4)
Unknown	23	17	74	21	16	11	13	9	184
**Site of infection**									
Urogenital	1163 (86.5)	1426 (84.7)	1466 (82.1)	1536 (83.0)	1531 (79.0)	1549 (76.3)	1496 (75.5)	1946 (75.5)	12,113 (79.7)
Anorectal	138 (10.3)	191 (11.4)	216 (12.1)	188 (10.2)	255 (13.2)	192 (9.5)	276 (13.9)	366 (14.2)	1822 (12.0)
Pharyngeal	34 (2.5)	59 (3.5)	79 (4.4)	92 (5.0)	122 (6.3)	154 (7.6)	178 (9.0)	165 (6.4)	883 (5.8)
Other	10 (0.7)	7 (0.4)	24 (1.3)	35 (1.9)	30 (1.5)	135 (6.7)	31 (1.6)	100 (3.9)	372 (2.5)
Unknown	21	83	117	75	56	121	54	86	613

*No* Number, *Hetero* Heterosexual, *MSM* Men who have sex with men, *UNK* Unknown sexual orientation

Briefly, the overall coverage of reporting gender, sexual orientation, and anatomical site of infection was 98.8% (range: 96.1–99.7%), 56.2% (range: 49.9–63.9%), and 96.1% (range: 93.8–98.5%), respectively. The isolates were mainly collected from males (*n* = 13,083, 83.8%; females *n* = 2536, 16.2%; gender not reported, *n* = 184). Of these patients, 3939 (25.2%), 3464 (22.2%), and 2536 (16.2%) reported sexual orientation as MSM, heterosexual males, and females, respectively. The isolates were cultured from urogenital samples (*n* = 12,113; 79.7%), anorectal samples (*n* = 1822; 12.0%), pharyngeal samples (*n* = 883; 5.8%), and samples from other anatomical sites (*n* = 372; 2.5%). The site of infection was not reported for 613 isolates (Table 1). Males who did not report as MSM and females were most likely to have urogenital infections, whereas MSM had the highest proportion of anorectal and pharyngeal infections (Table 2).

**Table 2:** Total number and proportion (%) of *Neisseria gonorrhoeae* isolates collected in Euro-GASP from 2009 to 2016 by gender and sexual orientation divided into the anatomical site of infection

Gender and sexual orientation	Urogenital No (%)	Anorectal No (%)	Pharyngeal No (%)	Other No (%)	UNK	Total
Females	2154 (88.2)	113 (4.6)	134 (5.5)	41 (1.7)	94	2536
Males_Hetero	3343 (97.4)	23 (0.7)	26 (0.8)	40 (1.2)	32	3464
Males_MSM	1928 (50.0)	1357 (35.2)	542 (14.1)	26 (0.7)	86	3939
Males_Other/UNK	4621 (86.0)	316 (5.9)	175 (3.2)	263 (4.9)	305	5680
Unknown	67	13	6	2	96	184
**Total**	12,113	1822	883	372	613	15,803

*No* Number, *Hetero* Heterosexual, *MSM* Men who have sex with men, *UNK* Unknown sexual orientation

### Antimicrobial resistance in Euro-GASP from 2009 to 2016 – association with gender and sexual orientation

**Fig. 1:**
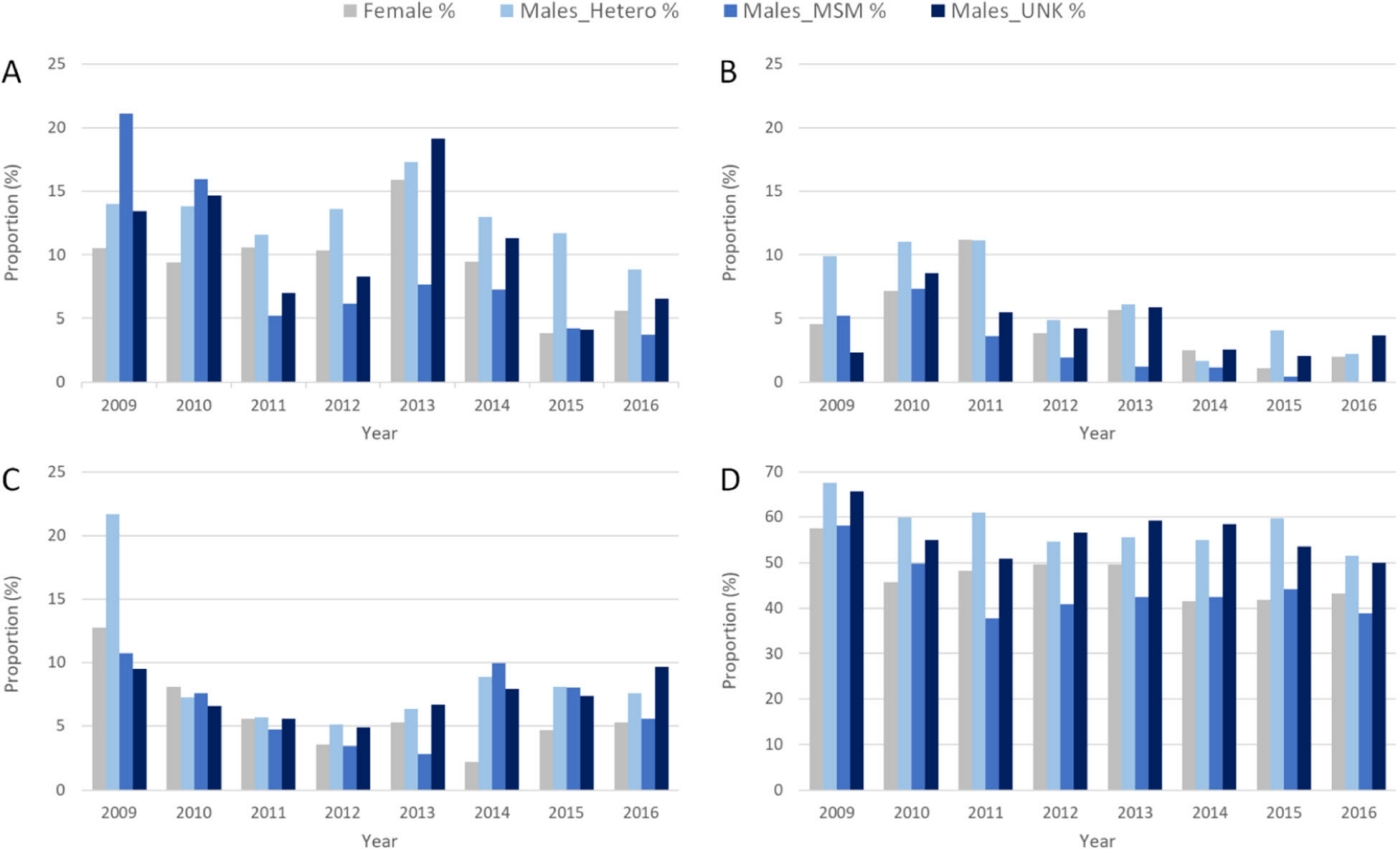
Proportion of *Neisseria gonorrhoeae* isolates with **a** resistance combined with decreased susceptibility to ceftriaxone, **b** resistance to cefixime, **c** resistance to azithromycin, and **d** resistance to ciprofloxacin, over time by gender and sexual orientation, 2009 to 2016

During 2009–2016, the overall resistance to ciprofloxacin, azithromycin, cefixime, and ceftriaxone was 51.7% (range during the years: 46.5–63.5%), 7.1% (4.5–13.2%), 4.3% (1.8–8.7%), and 0.2% (0.0–0.5%), respectively. The level of resistance combined with decreased susceptibility to ceftriaxone was 10.2% (5.7–15.5%). The proportion of isolates with resistance combined with decreased susceptibility to ceftriaxone, and resistance to cefixime, azithromycin and ciprofloxacin over time by gender and sexual orientation are shown in Fig. 1.

Despite that the overall level of resistance combined with decreased susceptibility to ceftriaxone was relatively high (10.2%), it decreased from 2009 to 2016 (Fig. 1a). Furthermore, the overall number of isolates with ceftriaxone resistance was low (*n* = 26, 0.2%) and none were collected from females. Rare isolates with ceftriaxone resistance initially emerged in 2011 (three isolates in Austria and seven in Germany) among five patients without gender or sexual orientation reported, four male patients with sexual orientation not reported, and one heterosexual male. Ceftriaxone resistance was subsequently identified in two MSM (Ireland and Slovenia) and one patient without gender or sexual orientation reported (Germany) in 2012, in six males with sexual orientation not reported (Spain) and one heterosexual male (Germany) in 2013, in three males with sexual orientation not reported (Germany, Greece, and Norway) and two heterosexual males (Greece) in 2014, and finally in one heterosexual male (Greece) in 2015 (no ceftriaxone resistance was found in 2016). The overall level of resistance combined with decreased susceptibility to ceftriaxone was 7.6% in MSM, 12.6% in heterosexual males, 10.6% in males with sexual orientation not reported, and 9.1% in females. Resistance combined with decreased susceptibility to ceftriaxone was significantly positively associated with isolates from heterosexual males, males with sexual orientation not reported, and females (*p* < 0.001, *p* < 0.001, and *p* = 0.025; ORs 1.76, 1.44, and 1.23, respectively). The level of gonococcal resistance to cefixime was 2.1, 6.0, 4.4, and 4.6% in MSM, heterosexual males, males with sexual orientation not reported, and females, respectively. Also cefixime resistance was significantly associated with isolates from heterosexual males, males with sexual orientation not reported, and females (all *p* < 0.001; ORs 3.0, 2.14, and 2.26). Regarding azithromycin, 6.5% of the isolates from MSM, 8.5% from heterosexual males, 7.3% from males with sexual orientation not reported, and 5.6% from females were resistant. Azithromycin resistance was significantly associated with heterosexual males (*p* < 0.001; OR 1.33). Finally, 43.2% of the isolates from MSM, 57.4% from heterosexual males, 56.0% from males with sexual orientation not reported, and 46.6% from females were resistant to ciprofloxacin. Ciprofloxacin resistance was significantly associated with heterosexual males, males with sexual orientation not reported, and females (*p* < 0.001, *p* < 0.001, and *p* = 0.007; ORs 1.78, 1.67, and 1.15). See supplementary tables S1, S2, S3 and S4 for all *p*-values, ORs and CIs.

### Antimicrobial resistance in Euro-GASP from 2009 to 2016 – association with anatomical site of infection

The number of Euro-GASP isolates collected from different anatomical sites and the number and proportion of isolates with resistance combined with decreased susceptibility to ceftriaxone, and resistance to cefixime, azithromycin and ciprofloxacin, by anatomical site is summarised in Table 3.

**Table 3:** *Neisseria gonorrhoeae* isolates in Euro-GASP from 2009 to 2016 by anatomical site of infection, and resistance combined with decreased susceptibility to ceftriaxone, and resistance to cefixime, azithromycin, and ciprofloxacin

Site of infection	Ceftriaxone			Cefixime		Azithromycin		Ciprofloxacin	
	No of tested	DS No (%)	R No (%)	No of tested	R No (%)	No of tested	R No (%)	No of tested	R No (%)
**Urogenital**	**12,113**	**1264 (10.4)**	**17 (0.1)**	**12,063**	**575 (4.8)**	**12,062**	**872 (7.2)**	**12,102**	**6508 (53.8)**
Female	2154	185 (8.6)	0 (0.0)	2154	96 (4.5)	2153	117 (5.4)	2153	993 (46.1)
Males_Hetero	3343	415 (12.4)	5 (0.1)	3307	196 (5.9)	3302	284 (8.6)	3343	1921 (57.5)
Males_MSM	1928	172 (8.9)	0 (0.0)	1917	57 (3.0)	1921	134 (7.0)	1926	900 (46.7)
Males_Other/UNK	4621	485 (10.5)	12 (0.3)	4618	222 (4.8)	4619	334 (7.2)	4613	2653 (57.5)
Unknown	67	7	0	67	4	67	3	67	41
**Anorectal**	**1822**	**149 (8.2)**	**1 (0.1)**	**1822**	**35 (1.9)**	**1822**	**118 (6.5)**	**1822**	**774 (42.5)**
Female	113	15 (13.3)	0 (0.0)	113	9 (8.0)	113	10 (8.9)	113	61 (54.0)
Males_Hetero	23	4 (17.4)	0 (0.0)	23	1 (4.4)	23	3 (13.0)	23	10 (43.5)
Males_MSM	1357	99 (7.3)	1 (0.1)	1357	18 (1.3)	1357	78 (5.8)	1357	556 (41.0)
Males_Other/UNK	316	29 (9.2)	0 (0.0)	316	6 (1.9)	316	26 (8.2)	316	140 (44.3)
Unknown	13	2	0	13	1	13	1	13	7
**Pharyngeal**	**883**	**72 (8.2)**	**2 (0.2)**	**883**	**22 (2.5)**	**883**	**73 (8.3)**	**883**	**365 (41.3)**
Female	134	20 (14.9)	0 (0.0)	134	10 (7.5)	134	14 (10.5)	134	66 (49.3)
Males_Hetero	26	2 (7.7)	0 (0.0)	26	2 (7.7)	26	0 (0.0)	26	16 (61.5)
Males_MSM	542	25 (4.6)	1 (0.2)	542	6 (1.1)	542	43 (7.9)	542	202 (37.3)
Males_Other/UNK	175	24 (13.7)	0 (0.0)	175	1 (0.6)	175	13 (7.4)	175	78 (44.6)
Unknown	6	1	1	6	3	6	3	6	3
**Other**	**372**	**22 (5.9)**	**0 (0.0)**	**372**	**13 (3.5)**	**372**	**22 (5.9)**	**372**	**187 (50.3)**
Female	41	3 (7.3)	0 (0.0)	41	1 (2.4)	41	1 (2.4)	41	16 (39.0)
Males_Hetero	40	5 (12.5)	0 (0.0)	40	4 (10.0)	40	2 (5.0)	40	26 (65.0)
Males_MSM	26	0 (0.0)	0 (0.0)	26	0 (0.0)	26	0 (0.0)	26	11 (42.3)
Males_Other/UNK	263	14 (5.3)	0 (0.0)	263	8 (3.0)	263	19 (7.2)	263	134 (51.0)
Unknown	2	0	0	2	0	2	0	2	0
**Unknown**	**613**	**76 (12.4)**	**6 (1.0)**	**611**	**27 (4.4)**	**613**	**33 (5.4)**	**613**	**323 (52.7)**
Female	94	9 (9.6)	0 (0.0)	93	1 (1.1)	94	1 (1.1)	94	45 (47.9)
Males_Hetero	32	7 (21.9)	0 (0.0)	32	3 (9.4)	32	3 (9.4)	32	17 (53.1)
Males_MSM	86	1 (1.2)	0 (0.0)	86	1 (1.2)	86	2 (2.3)	86	31 (36.1)
Males_Other/UNK	305	35 (11.5)	1 (0.3)	304	10 (3.3)	305	23 (7.5)	305	172 (56.4)
Unknown	96	24	5	96	12	96	4	96	58
**Total**	**15,803**	**1583 (10.0)**	**26 (0.2)**	**15,751**	**672 (4.3)**	**15,752**	**1118 (7.1)**	**15,792**	**8157 (51.7)**

*No* Number, *R* Resistance, *DS* Decreased susceptibility, *UNK* Unknown sexual orientation

The majority (79.3%) of AMR isolates (resistant to at least one antimicrobial) were cultured from urogenital samples, reflecting that most (76.7%) Euro-GASP isolates during 2009 to 2016 were obtained from urogenital sites.

Where site of infection was known for ceftriaxone-resistant isolates (20/26), all were from urogenital site (*n* = 17), with exception of two pharyngeal isolates (2012, Germany and Slovenia (MSM)) and one rectal isolate from Ireland (2012, MSM). Overall, resistance combined with decreased susceptibility to ceftriaxone was found in 10.6% of the isolates from urogenital site, 8.2% from anorectal, and 8.4% from pharyngeal. Resistance combined with decreased susceptibility to ceftriaxone was significantly associated with urogenital samples compared to anorectal and pharyngeal samples (*p* = 0.002, *p* = 0.039; ORs 0.76 and 0.77). The level of resistance to cefixime was 4.8, 1.9, and 2.5% in urogenital, anorectal, and pharyngeal isolates, respectively, and also significantly associated with urogenital samples compared to anorectal and pharyngeal samples (*p* < 0.001, *p* = 0.002; ORs 0.39 and 0.51). For azithromycin 7.2% of the isolates from urogenital, 6.5% from anorectal, and 8.3% from pharyngeal sites were resistant; with no significant association to any anatomical site of infection. Finally, 53.8% of the isolates from urogenital, 42.5% from anorectal, and 41.3% from pharyngeal sites were resistant to ciprofloxacin (Table 3). Ciprofloxacin resistance was significantly associated with isolates from urogenital site when compared to anorectal and pharyngeal samples (both *p* < 0.001; ORs 0.63 and 0.61, respectively). See supplementary tables S1, S2, S3 and S4 for all *p*-values, ORs and CIs.

**Fig. 2:**
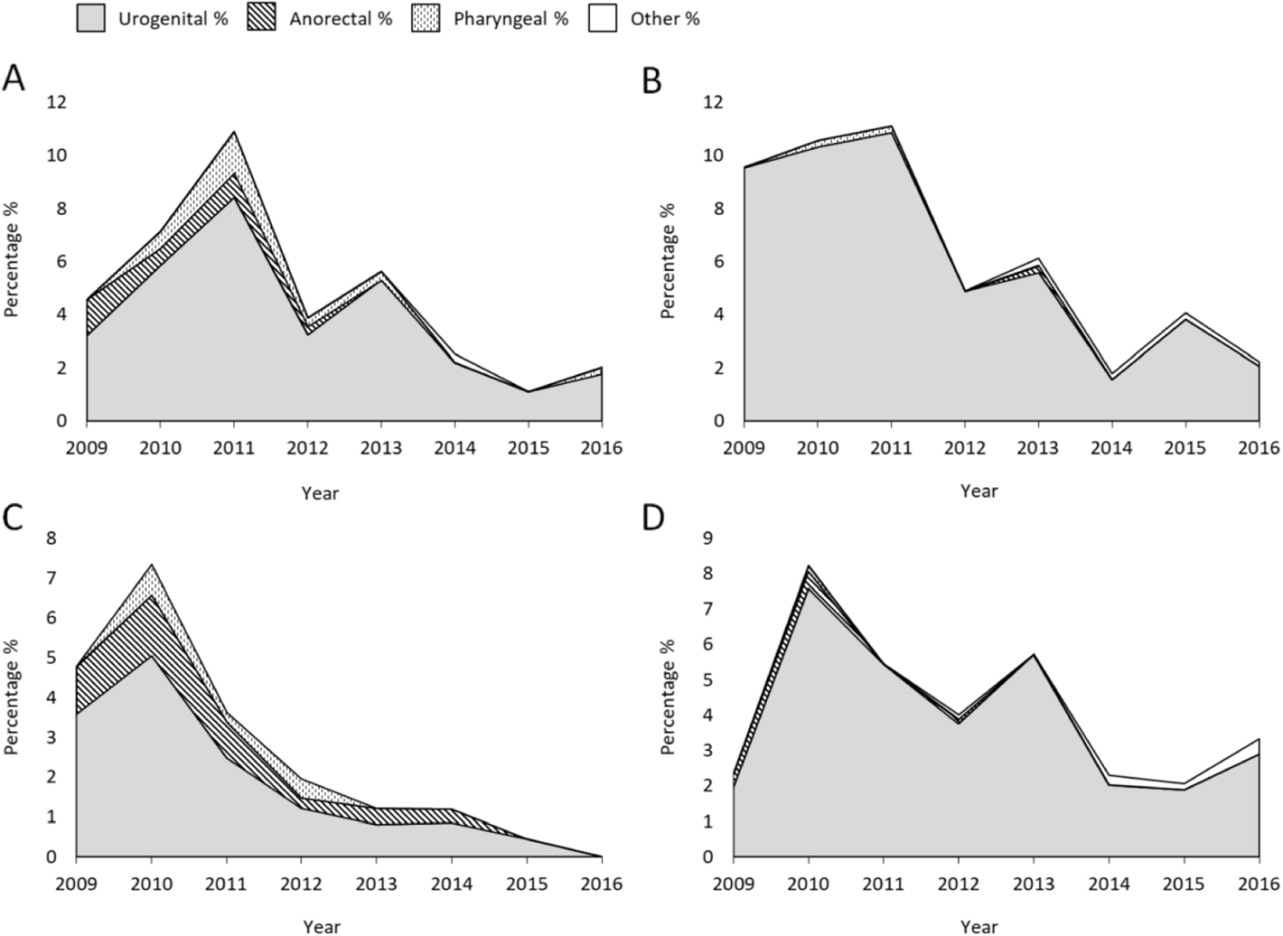
Proportion of cefixime-resistant gonococcal isolates divided into gender, sexual orientation, and anatomical site of infection displayed over time. **a** Females **b** Heterosexual males **c** Men who have sex with men (MSM), and **d** Males with other sexual orientation or not reported

When combining gender, sexual orientation and anatomical site of infection over time, cefixime resistant isolates were mostly from the urogenital site. However, during the overall increase in 2010 and 2011 the proportion of anorectal and pharyngeal isolates increased by primarily anorectal isolates among MSM in 2010 and subsequently pharyngeal isolates among females in 2011 (Fig. 2).

**Fig. 3:**
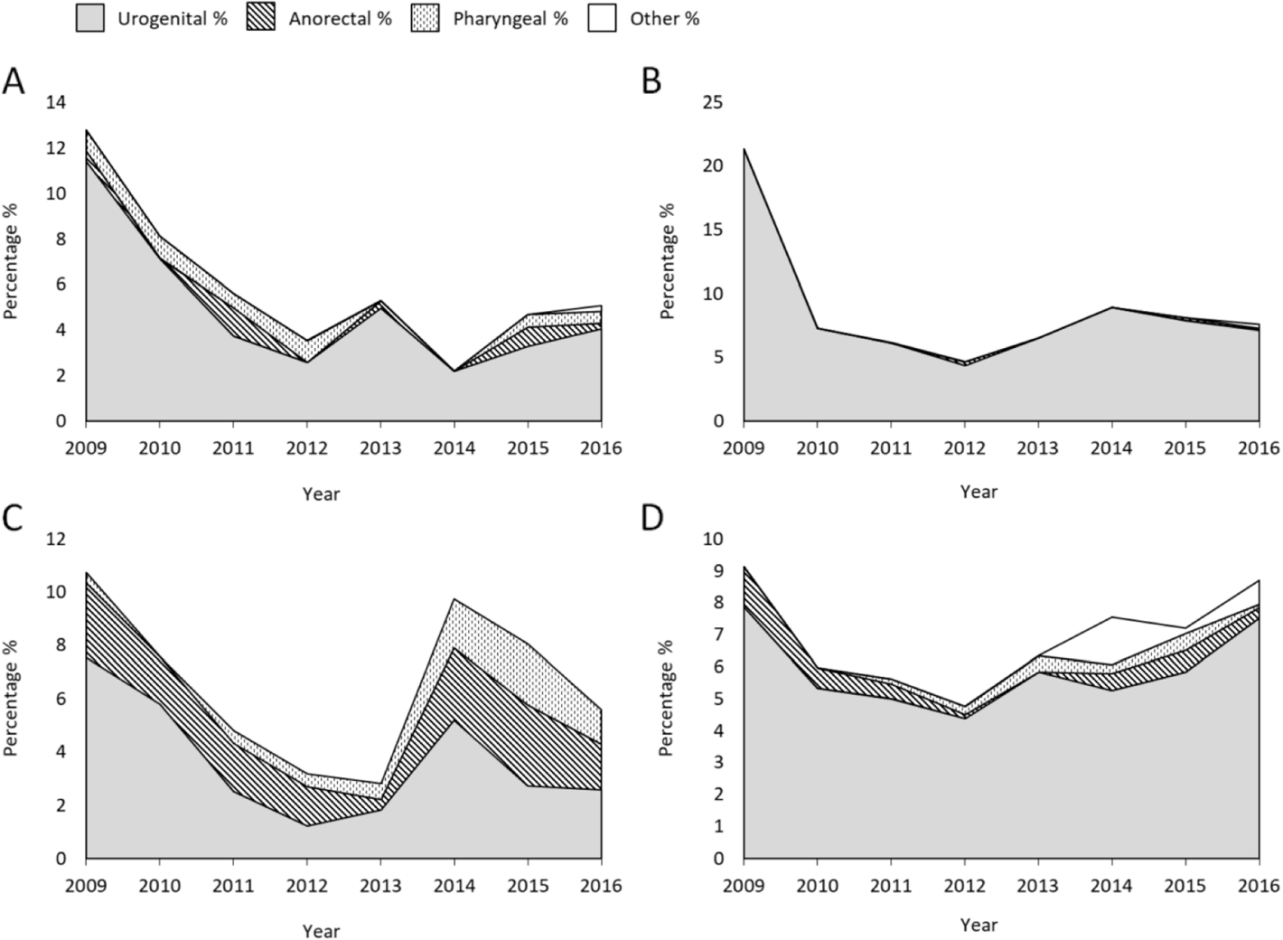
Proportion of azithromycin-resistant gonococcal isolates divided into gender, sexual orientation and anatomical site of infection displayed over time. **a** Females **b** Heterosexual males **c** Men who have sex with men (MSM), and **d** Males with other sexual orientation or not reported

Also azithromycin-resistant isolates were most frequently from urogenital site, however, the proportion of anorectal and pharyngeal isolates increased in MSM in 2014, and a small increase of anorectal isolates was seen in females in 2015 (Fig. 3).

**Fig. 4:**
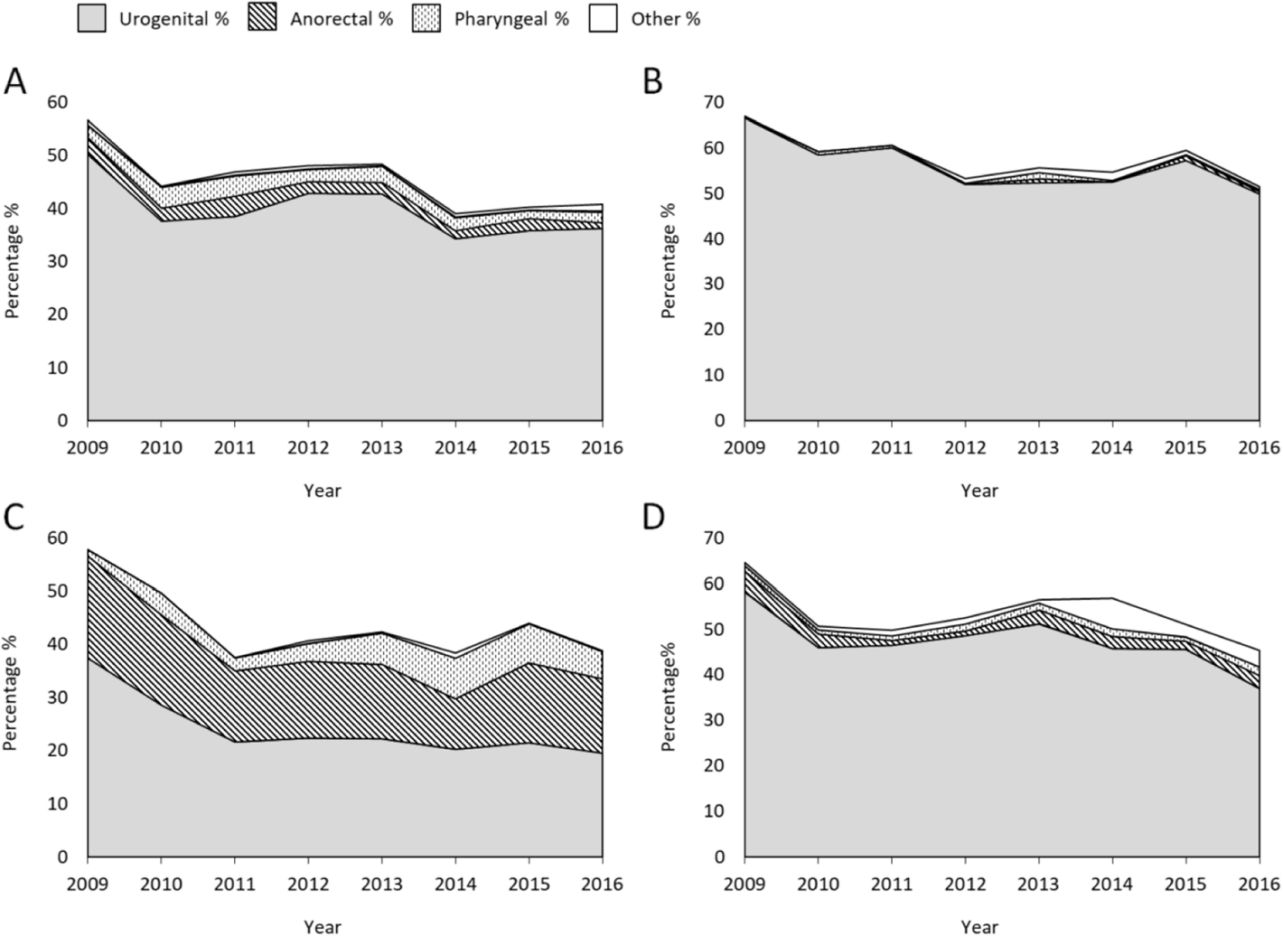
Proportion of ciprofloxacin-resistant gonococcal isolates divided into gender, sexual orientation and anatomical site of infection displayed over time. **a** Females **b** Heterosexual males **c** Men who have sex with men (MSM), and **d** Males with other sexual orientation or not reported

Ciprofloxacin resistance was relatively high and stable from 2009 to 2016 and mostly identified in urogenital isolates, but also in anorectal isolates from MSM (Fig. 4).

Finally, most of the statistical associations reported above remained valid in the multivariate analysis in respect to cefixime and ciprofloxacin resistance/susceptibility and gender, sexual orientation, and site of infection with the exception of the association between ciprofloxacin resistance and females (supplementary tables S1, S2, S3 and S4). Ceftriaxone resistance combined with decreased susceptibility remained associated with non-MSM males, as well as unknown site of infection. When controlling for year, there was no longer an association between cefixime susceptibility and pharyngeal infections. All other azithromycin, ceftriaxone and ciprofloxacin associations remained when controlling for year in a multivariate analysis.

## Discussion

From 2009 to 2016, the overall resistance to ceftriaxone among the Euro-GASP isolates (*n* = 15,803) was only 0.2% (0.0–0.5% during the years), however, resistance combined with decreased susceptibility to ceftriaxone was 10.2% (5.7–15.5%). The overall resistance to cefixime, azithromycin, and ciprofloxacin was 4.3% (1.8–8.7%), 7.1% (4.5–13.2%), and 51.7% (46.5–63.5%), respectively. Resistance combined with decreased susceptibility to ceftriaxone, resistance to cefixime, and resistance to ciprofloxacin were all significantly associated with heterosexual males, males with sexual orientation not reported, and females (except for ciprofloxacin), i.e. when compared to MSM. Notably, none of the 26 ceftriaxone-resistant isolates in 2009–2016 was cultured from a female. Resistance to azithromycin was significantly associated with heterosexual males compared to MSM. Furthermore, resistance combined with decreased susceptibility to ceftriaxone, resistance to cefixime, and resistance to ciprofloxacin were all significantly associated with urogenital site of infection, while no significant association with site of infection was observed for azithromycin. Overall, it appeared that sexual orientation was the main variable associated with AMR, which resulted in some associations also with urogenital site of infection because these infections were more common among heterosexual patients, while anorectal and pharyngeal infections were more common among MSM. Nevertheless, the levels of AMR in all urogenital and extragenital sites were substantial and extragenital infections were relatively common also in females. Accordingly, diagnostic 3-site testing (urogenital, pharyngeal, and anorectal sites) for *N. gonorrhoeae* is of obvious importance, i.e. to limit the spread of infection as well as to detect gonococcal AMR. This 3-site testing should ideally use molecular diagnostics for the extragenital samples and be routine in MSM, considered in women particularly if sexual contacts of gonorrhoea patients, and be guided based on sexual history, risk, travel history and symptoms or signs in all other patients [8, 12, 52–54].

Previous studies from the Gonococcal Isolate Surveillance Project (GISP) in the USA have shown significantly higher geometric mean MICs of azithromycin in MSM than heterosexual males [55, 56]. However, in Euro-GASP we could not find the same association with azithromycin resistance or geometric mean (data not shown), and instead azithromycin resistance was significantly associated with heterosexual males. Nevertheless, it cannot be excluded that the low coverage on reporting on the epidemiological variable sexual orientation underestimates the proportion of MSM. This could potentially bias our associations, but this bias was considered limited because males with sexual orientation not reported were not significantly associated with azithromycin resistance. A feasible explanation to the higher prevalence of azithromycin resistance in males than females might be the frequent use of azithromycin 1 g to treat male non-gonococcal urethritis and *Chlamydia trachomatis* infections. Also in contrast to our findings, studies from the US GISP and the Gonococcal Resistance to Antimicrobials Surveillance Program (GRASP) in England and Wales have reported that ceftriaxone and cefixime resistance is strongly associated with MSM [56–58]. In Euro-GASP, this association with MSM was observed in earlier years, i.e. in 2009–2010 [59], however, in 2013 cefixime resistance and decreased susceptibility to ceftriaxone was more strongly associated with heterosexual patients [27]. In line with this, the initial wider spread of ciprofloxacin resistance documented in US GISP as well as in GRASP was predominantly among MSM, but then over time was bridged over to heterosexual males and females [34, 35, 60, 61]. A similar initial emergence and spread of ciprofloxacin resistance in many EU/EEA countries cannot be excluded.

In general, larger studies regarding differences in gonococcal antimicrobial susceptibility in different anatomical sites in both genders are extremely limited and nearly absent, which is especially because most larger gonococcal AMR surveillance programmes have predominantly or only collected urogenital samples in males (US GISP). However, it has been suggested that pharyngeal gonococcal infection is an asymptomatic reservoir for AMR and especially initial emergence of AMR [38–40]. Furthermore, it has also been hypothesized that gonococcal isolates with *mtrR* mutations, which today exist in the majority of circulating gonococcal isolates, may have enhanced survival in rectum and potentially can act as a reservoir for azithromycin resistance in especially MSM [62]. However, our present data did not support that isolates from the pharynx or anorectum were more resistant to any tested antimicrobial, as has been described in previous studies [43, 44, 46]. In contrast, overall resistance combined with decreased susceptibility to ceftriaxone, cefixime resistance, and ciprofloxacin resistance was significantly associated with urogenital site of infection.

The main limitation of the present study was that all analysis was performed on the entire Euro-GASP material, which includes samples of consecutive gonorrhoea patients and gonococcal isolates from many diverse countries [6, 7, 27–29, 47–50]. The gonorrhoea epidemiology, AMR levels, and level of reporting, especially in regard to sexual orientation, can differ in the countries participating in the Euro-GASP. However, country-by-country analysis was not possible to perform due to the lack of sufficient statistical power when analyzing the relatively small annual Euro-GASP samples from individual countries. We encourage similar studies to be performed on national level, analyzing all gonorrhoea cases and gonococcal isolates in the countries. Furthermore, the large proportion of urogenital isolates compared to rectal and pharyngeal isolates was an additional limitation.

## Conclusions

In the light of the recent treatment failures, mainly of pharyngeal gonorrhoea, and the potential onward transmission of ceftriaxone-resistant gonococcal isolates, antimicrobial susceptibility surveillance as well as verification and reporting of treatment failures should be enhanced, and actions to develop alternative gonorrhoea therapeutic antimicrobials and treatment strategies are essential [63, 64]. In the present study, sexual orientation was the main variable associated with gonococcal AMR. Unexpectedly, the strongest associations with AMR were identified with heterosexual patients, particularly males, and not MSM. To provide evidence-based understanding and mitigate gonococcal AMR emergence and spread, associations between antimicrobial susceptibility/resistance and patients’ gender, sexual orientation and anatomical site of infection, as well as individual-level behavioural risk factors, need to be further investigated in different geographic settings. In general, these insights will support identification of risk groups and targeted public health actions such as intensified screening, 3-site testing using molecular diagnostics to more effectively identify also pharyngeal and anorectal gonorrhoea, sexual contact tracing, and enhanced surveillance of culture-based AMR and treatment failures.

## Supplementary Information

**Additional file 1: Table S1.** Univariate association of ceftriaxone resistance (R) combined with decreased susceptibility (DS) or susceptibility and patient characteristics, Euro-GASP, 2009–2016. **Table S2.** Univariate association of cefixime resistance/susceptibility and patient characteristics, Euro-GASP, 2009–2016. **Table S3.** Univariate association of azithromycin resistance/susceptibility and patient characteristics, Euro-GASP, 2009–2016. **Table S4.** Univariate association of ciprofloxacin resistance/susceptibility and patient characteristics, Euro-GASP, 2009–2016.

## Data Availability

The data that support the findings of this study are available from the ECDC, but restrictions apply to the availability of these data, which were used under license for the current study, and so are not publicly available. Data may however be available from the authors upon reasonable request and with permission of the ECDC.

## Abbreviations

AMR: Antimicrobial resistance
CI: Confidence interval
DS: Decreased susceptibility
ECDC: European Centre for Disease Prevention and Control
EEA: European Economic Area
ESC: Extended-spectrum cephalosporin
EU: European Union
EUCAST: European Committee on Antimicrobial Susceptibility Testing
Euro-GASP: European Gonococcal Antimicrobial Surveillance Programme
GISP: Gonococcal Isolate Surveillance Project
GRASP: Gonococcal Resistance to Antimicrobials Surveillance Programme
Hetero: Heterosexual
I: Susceptibility, increased exposure
MIC: Minimum inhibitory concentration
MSM: Men who have sex with men
No: Number
R: Resistance
S: Susceptibility
TESSy: The European Surveillance System
UNK: Unknown
