# Supplementary material for: Associations between antimicrobial susceptibility/resistance of Neisseria gonorrhoeae isolates in European Union/European Economic Area and patients’ gender, sexual orientation and anatomical site of infection, 2009–2016
Source: BMC Infect Dis. 2021 Mar 18;21:273. doi: 10.1186/s12879-021-05931-0 (PMC7976712; doi:10.1186/s12879-021-05931-0)
Supplement: Supplementary file 1 — Additional file 1: Table S1. Univariate association of ceftriaxone resistance (R) combined with decreased susceptibility (DS) or susceptibility and patient characteristics, Euro-GASP, 2009–2016. Table S2. Univariate association of cefixime resistance/susceptibility and patient characteristics, Euro-GASP, 2009–2016. Table S3. Univariate association of azithromycin resistance/susceptibility and patient characteristics, Euro-GASP, 2009–2016. Table S4. Univariate association of ciprofloxacin resistance/susceptibility and patient characteristics, Euro-GASP, 2009–2016. [file 12879_2021_5931_MOESM1_ESM.docx]

**Associations between antimicrobial susceptibility/resistance of *Neisseria gonorrhoeae* isolates in European Union/European Economic Area and patients’ gender, sexual orientation and anatomical site of infection, 2009-2016**

Susanne Jacobsson^1ǂ^, Michelle J. Cole^2ǂ^, Gianfranco Spiteri^3^, Michaela Day^2^, and Magnus Unemo^1*^, on behalf of The Euro-GASP Network

^ǂ^Joint first authors

*Correspondence: magnus_unemo@yahoo.com

^1^WHO Collaborating Centre for Gonorrhoea and other STIs, National Reference Laboratory for Sexually Transmitted Infections, Department of Laboratory Medicine, Faculty of Medicine and Health, Örebro University, Örebro, Sweden. ^2^National Infection Service, Public Health England, Colindale, United Kingdom. ^3^European Centre for Disease Prevention and Control, Stockholm, Sweden.

Supplementary tables

Table S1 Univariate association of ceftriaxone resistance (R) combined with decreased susceptibility (DS) or susceptibility and patient characteristics, Euro-GASP, 2009-2016

|  | **Ceftriaxone R+DS**  **N (%)** | **Odds ratio** | **95% CI** | **P value** | **P value - Multivariate** |
| --- | --- | --- | --- | --- | --- |
| **Site of infection (N=15190)** |  |  |  |  |  |
| Genital (12113) | 1281 (10.6%) | 1 |  |  |  |
| Anorectal (1822) | 150 (8.2%) | 0.76 | 0.64-0.91 | **0.002** | 0.579 |
| Pharyngeal (883) | 74 (8.4%) | 0.77 | 0.61-0.99 | **0.039** | 0.515 |
| Other (372) | 22 (5.9%) | 0.53 | 0.34-0.82 | **0.004** | **0.005** |
| **Sexual orientation & gender (N=15619)** | |  |  |  |  |
| MSM (3939) | 299 (7.6%) | 1 |  |  |  |
| Male heterosexual (3464) | 438 (12.6%) | 1.76 | 1.51-2.06 | **<0.001** | **<0.001** |
| Males UNK/Other (5680) | 600 (10.6%) | 1.44 | 1.24-1.66 | **<0.001** | **<0.001** |
| Female (2536) | 232 (9.1%) | 1.23 | 1.03-1.47 | **0.026** | 0.101 |

N, number; CI, confidence interval; MSM, men who have sex with men; UNK, unknown

Table S2 Univariate association of cefixime resistance/susceptibility and patient characteristics, Euro-GASP, 2009-2016

|  | **Cefixime resistance N (%)** | **Odds ratio** | **95% CI** | **P value** | **Adjusted ORs (95% CI)- Multivariate** | **P value - Multivariate** |
| --- | --- | --- | --- | --- | --- | --- |
| **Site of infection (N=15140)** |  |  |  |  |  |  |
| Genital (12063) | 575 (4.8%) | 1 |  |  |  |  |
| Anorectal (1822) | 35 (1.9%) | 0.39 | 0.28-0.55 | **<0.001** | **0.58 (0.40-0.85)** | **0.005** |
| Pharyngeal (883) | 22 (2.5%) | 0.51 | 0.33-0.79 | **0.002** | **0.61 (0.38-0.98)** | **0.042** |
| Other (372) | 13 (3.5%) | 0.72 | 0.41-1.27 | 0.255 | 0.74 (0.41-1.30) | 0.295 |
| **Sexual orientation & gender (N=15619)** | |  |  |  |  |  |
| MSM (3939) | 82 (2.1%) | 1 |  |  |  |  |
| Male heterosexual (3464) | 206 (6.0%) | 3.0 | 2.31-3.90 | **<0.001** | **2.37 (1.78-3.15)** | **<0.001** |
| Males UNK/Other (5680) | 247 (4.4%) | 2.14 | 1.66-2.75 | **<0.001** | **1.80 (1.36-2.36)** | **<0.001** |
| Female (2536) | 117 (4.6%) | 2.26 | 1.70-3.02 | **<0.001** | **1.93 (1.42-2.620** | **<0.001** |

N, number; CI, confidence interval; ORs, odds ratios; MSM, men who have sex with men; UNK, unknown

Table S3 Univariate association of azithromycin resistance/susceptibility and patient characteristics, Euro-GASP, 2009-2016

|  | **Azithromycin resistance N (%)** | **Odds ratio** | **95% CI** | **P value** | **P value - Multivariate** |
| --- | --- | --- | --- | --- | --- |
| **Site of infection (N=15139)** |  |  |  |  |  |
| Genital (12062) | 872 (7.2%) | 1 |  |  |  |
| Anorectal (1822) | 118 (6.5%) | 0.89 | 0.73-1.08 | 0.244 | **n/a** |
| Pharyngeal (883) | 73 (8.3%) | 1.16 | 0.90-1.48 | 0.252 |  |
| Other (372) | 22 (5.9%) | 0.81 | 0.52-1.25 | 0.333 |  |
| **Sexual orientation & gender (N=15619)** | |  |  |  |  |
| MSM (3939) | 257 (6.5%) | 1 |  |  |  |
| Male heterosexual (3464) | 292 (8.4%) | 1.33 | 1.12-1.59 | **0.001** |  |
| Males UNK/Other (5680) | 415 (7.3%) | 1.12 | 0.96-1.32 | 0.153 |  |
| Female (2536) | 143 (5.6%) | 0.86 | 0.70-1.06 | 0.160 |  |

N, number; CI, confidence interval; MSM, men who have sex with men; UNK, unknown

Table S4 Univariate association of ciprofloxacin resistance/susceptibility and patient characteristics, Euro-GASP, 2009-2016

|  | **Ciprofloxacin resistance N (%)** | **Odds ratio** | **95% CI** | **P value** | **Adjusted ORs (95% CI)- Multivariate** | **P value - Multivariate** |
| --- | --- | --- | --- | --- | --- | --- |
| **Site of infection (N=15179)** |  |  |  |  |  |  |
| Genital (12102) | 6508 (53.8%) | 1 |  |  |  |  |
| Anorectal (1822) | 774 (42.5%) | 0.63 | 0.58-0.70 | **<0.001** | **0.78 (0.69-0.87)** | **<0.001** |
| Pharyngeal (883) | 365 (41.3%) | 0.61 | 0.53-0.70 | **<0.001** | **0.73 (0.63-0.84)** | **<0.001** |
| Other (372) | 187 (50.3%) | 0.87 | 0.71-1.07 | 0.182 | 0.83 (0.67-1.02) | 0.080 |
| **Sexual orientation & gender (N=15619)** | |  |  |  |  |  |
| MSM (3939) | 1700 (43.2%) | 1 |  |  |  |  |
| Male heterosexual (3464) | 1990 (57.4%) | 1.78 | 1.62-1.95 | **<0.001** | **1.56 (1.40-1.73)** | **<0.001** |
| Males UNK/Other (5680) | 3177 (55.9%) | 1.67 | 1.54-1.82 | **<0.001** | **1.51 (1.37-1.65)** | **<0.001** |
| Female (2536) | 1181(46.6%) | 1.15 | 1.04-1.27 | **0.007** | 1.03 (0.92-1.15) | 0.624 |

N, number; CI, confidence interval; MSM, men who have sex with men; UNK, unknown
